# The MILEPOST Framework for Integrating mHealth Into Community-Driven Interventions: Lessons Learned From a Human-Centered Design Approach in Rural Uganda

**DOI:** 10.9745/GHSP-D-24-00061

**Published:** 2026-08-10

**Authors:** Josephine Schwab, Jonas Wachinger, Maxencia Nabiryo, Juliette Cazier, Juliet Wanyana, Kizito Mayombwe, Ezekiel Okou, Dennis Oloka, John Bosco Ntambara, Richard Munana, Ivan Weswa, Isaac Sekitoleko, Andrew Basenero, Rebecca Ingenhoff, Caterina Favaretti, Vasanthi Subramonia Pillai, Till Bärnighausen, Nikkil Sudharsanan, Robert Kalyesubula, Christine Nalwadda, Shannon A. McMahon

**Affiliations:** a Heidelberg Institute of Global Health, Faculty of Medicine and University Hospital, Heidelberg University, Im Neuenheimer Feld 672, 69120, Heidelberg, Germany; b Department of Community Health and Behavioral Sciences, School of Public Health, College of Health Sciences, Makerere University, Mulago Hill Road, Kampala, 7072, Uganda; c mTOMADY GmbH, c/o Berlin Institute of Health, Bertolt-Brecht-Platz 3, 10117, Berlin, Germany; d African Community Center for Social Sustainability, Kiwembe Zone, Nakaseke Town Council, Nakaseke District, 28993, Uganda; e Charité Center for Global Health, Charité – Universitätsmedizin Berlin, Berlin, Germany; f Professorship of Behavioral Science for Disease Prevention and Health Care, Technical University of Munich, Am Olympiacampus 11, 80809, Munich, Germany; g Munich Center for Health Economics and Policy (M-CHEP), Munich, Germany; h Department of Global Health and Population, Harvard T.H. Chan School of Public Health, Harvard University, Boston, USA; i Africa Health Research Institute (AHRI), Somkhele and Durban, South Africa; j Department of Physiology, College of Health Sciences, Kampala, Uganda

## Abstract

Participatory design approaches to develop global health interventions have gained traction in recent years. However, while broad frameworks for the application of design approaches exist, literature outlining concrete experiences of and guidance for navigating specific design challenges remains limited. This article proposes a design framework based on our own experiences when applying human-centered design principles to bolster sustainable hypertension medication financing in rural Uganda. In our case study, we embedded an mHealth platform within a community-led health intervention aiming to fund blood pressure medication through a shared financial pooling system. Over the course of this project and in close collaboration with intervention end-users, we developed a design framework that outlines challenges, decision-making processes, and design solutions. We iteratively refined the framework between November 2021 and April 2023 over two phases of extensive formative research and one phase of structured qualitative data collection, consisting of 55 in-depth interviews and 4 focus group discussions with clients with hypertension, health care providers, and key intervention stakeholders. The resulting MILEPOST framework consists of 7 domains to consider amid mHealth intervention co-development: medical (e.g., health challenge scope and management); interpersonal (e.g., communication, decision-making, and trust within and across communities); logistical (e.g., current processes and sustainable implementation pathways); ethical (e.g., research and implementation ethics); political (e.g., stakeholder buy-in and long-term support); scientific (e.g., rigorous and feasible approaches for data collection, management, and analysis); and technical (e.g., mHealth component development, refinement, and implementation). We define each domain, provide examples of design challenges and derived solutions as they emerged in our work, and outline starting points for operationalizing each domain in other contexts or studies. We discuss how MILEPOST can provide guidance to researchers and implementors by contributing specific and actionable insights for participatory health intervention design efforts.

## Abstract (precis)

Based on human-centered design work in Uganda, we introduce the MILEPOST framework for developing and implementing impactful co-designed mHealth interventions, comprising 7 essential design domains: medical, interpersonal, logistical, ethical, political, scientific, and technical.

## Teaser

**Key Findings:**

- We outline design experiences from working with clients and key stakeholders in rural Uganda, where we built on a community initiative and co-developed an mHealth intervention for sustainable hypertension medication financing.
- We propose that teams consider medical, interpersonal, logistical, ethical, political, scientific, and technical design domains (referred to as the MILEPOST framework) in an iterative and non-linear manner to facilitate mHealth intervention acceptability and uptake.

**Key Implications:**

- We provide a checklist, including key questions to ask and stakeholders to involve for each MILEPOST domain, which can guide researchers, designers, and implementors in applying MILEPOST within their own work.
- We call on global health researchers to acknowledge potential synergies and tensions between design domains, especially when scientific considerations might not fully align with setting-specific routines or requirements.
- We encourage intervention designers and implementors to be sensitive to the broad spectrum of design challenges emerging in their contexts and to modify the MILEPOST domains based on their settings or experiences.
- We encourage sharing design experiences with scientific audiences, including experiences with and adaptations of the MILEPOST framework, to inform efforts across various design landscapes and priorities.

## INTRODUCTION

Design and implementation of global health interventions require careful consideration of broader medical and sociocultural contexts to facilitate intervention effectiveness, acceptability, and uptake.^1^ The systematic assessment and integration of these factors throughout the design process can help avoid decontextualized and ultimately inefficient interventions.^2^ Consequently, approaches acknowledging insights from end-users (including clients, health care providers, policymakers, or other stakeholders) have gained traction in global health work in recent years.^3^

One increasingly applied approach for the structured and iterative development of health interventions is human-centered design (HCD),^4^ often conceptualized as comprising 5 phases: Empathize, Define, Ideate, Prototype, and Test.^5^ As part of this process, HCD emphasizes the involvement of end-users throughout design and implementation stages to co-develop novel and acceptable solutions. Recent successful applications of HCD approaches for intervention design include vaccine promotion efforts,^6^ decision-making mechanisms in emergency medicine,^7^ and health care provision for noncommunicable diseases (NCDs).^8^^,^^9^

As the list of successful interventions developed using HCD expands, global health researchers have proposed, adopted, and refined a broad array of general and context-specific design frameworks to guide intervention development and implementation.^10^^,^^11^ While these frameworks form an integral resource to guide design processes in global health research and practice, they often focus on iteration and evaluation phases, as well as on broader, more abstract design domains to ensure generalizability across fields of application.

As a result, literature on how to bridge the gap between broadly applicable frameworks and specific challenges emerging during on-the-ground design work remains limited. In light of increasing evidence that HCD-based efforts might struggle with the sustainable implementation of derived solutions,^12^ along with a growing emphasis on the need for rigorous scientific methods in the design and evaluation of interventions,^13^ such practical guidance could provide starting points for navigating contextual specificities and methodological challenges within broader design efforts.

To contribute toward filling this gap in the literature, we applied HCD principles to iteratively develop and refine an mHealth intervention for community-based hypertension medication financing. In this article, we draw on our project experiences to outline a framework for conceptualizing 7 key design domains to be considered over the course of a meaningful co-creation process. We hope to inspire other researchers and practitioners aiming to design and integrate mHealth interventions tailored to specific contexts and stakeholders.

## METHODS

### Context and Setting

The focus of the overarching co-design research project^14^ was to leverage HCD approaches and mHealth technologies to explore the potential of building on—and digitizing—an existing community-led program for improving hypertension medication access in rural Uganda. Uganda, similar to many other low- and middle-income countries, faces a disproportionately high burden of NCDs, especially cardiovascular diseases.^15^ This burden is expected to further increase in the future, as 94.7% of the Ugandan population present one or more elevated risk factors for CVDs,^16^ especially hypertension.^17^^,^^18^

Given a broad array of challenges for sustainable access to hypertension treatment in the country, including at individual, structural, and political levels,^19^ a nongovernmental organization (ACCESS Uganda), together with clients with hypertension and health care providers, initiated a community-based medication financing scheme in rural Nakaseke district. The aim of our HCD-based study was to build on this initiative by co-creating an intervention prototype that formalized monetary flows and facilitated accountability and follow-up, incorporating mobile money transactions to minimize safety concerns. For a detailed description of the case study, the community-led initiative, and the co-created prototype, see the Box.

BOX Co-Development of the mHealth Intervention Prototype to Facilitate Hypertension Medication Financing in Rural Uganda

In Nakaseke, a district in central Uganda, an estimated 34.3% of the population aged 18 to 69 have hypertension.^50^ Despite antihypertensive medication being officially available free of cost at government health facilities, frequent stock-outs prevent routine medication access.^19^ This challenge is particularly acute for small-scale farmers and low-income households, including most of Nakaseke’s population,^51^ who often cannot afford the out-of-pocket costs of purchasing medication at private pharmacies.^19^
In this setting, a local nongovernmental organization, ACCESS Uganda, partnered with Semuto Health Center IV, a public outpatient clinic providing essential health care services for clients within a radius of roughly 15 km, to organize weekly NCD care days. Clients with hypertension could visit on these designated care days to receive health education and treatment in line with government mandates, including free medication when available. To address frequent medication stock-outs, facility clients, health care providers, and representatives of ACCESS Uganda developed a paper-based community-driven financing program. In this program, clients could contribute money to an ACCESS-based pool, which was used to purchase clinic supplies and antihypertensive medication in bulk. This end-user-initiated program, while appreciated by the community, proved logistically difficult to maintain due to excessive paperwork and challenges to reliably account for contributions or safely manage cash transactions at facility level.
To build on this initiative, we conducted the IMPEDE CVD study^14^ between 2021 and 2024 based on HCD principles. Initial parameters, as outlined in the funded project proposal and the published protocol paper,^14^ included the broad health challenge to be addressed (NCDs, with a likely focus on hypertension) and the aim to explore the suitability of an mHealth component. In the early phases of the project, members of ACCESS Uganda identified Semuto Health Center IV as a suitable setting due to the preexisting engagement and community-initiated saving program. The overarching study included two phases of formative and qualitative data collection, as well as a 6-month randomized controlled trial during which the intervention was tested. The broader study team included social scientists, medical professionals, NCD and design researchers, and software developers from Ugandan and German institutions.
The final intervention prototype, called MoPuleesa, draws on a client- and provider-facing mHealth component, combined with facility-based structures for usability support and medication distribution. Through a pooled-account structure, clients can contribute funds using mobile wallets. The pooled funds are then used by an ACCESS representative to purchase medication in bulk at wholesale prices to fill gaps in government medication supplies. Utilizing the benefits of a digital platform and mobile money transfers, the intervention intended to foster trust in reliable medication availability, thereby facilitating medication adherence and satisfaction with medical care (Supplement 1).
Over the course of pilot implementation, the intervention prototype was well-received by end-users and health system stakeholders, including coverage on national television, sparking interest in extending its application within the region.^52^^,^^53^ The pilot phase established intervention feasibility and usability among respondents within the intended setting and led to continuous and sustained improvements in medication availability.^54^ Additionally, ongoing implementation suggests longer-term sustainability. After external project funding concluded in 2023, ACCESS Uganda, in partnership with the community, continued to refine and maintain the intervention prototype and upscaled the intervention to include a private health facility in the region with similar dedicated NCD treatment days. As of this writing (May 2026), the intervention is ongoing at this private health facility and continues to generate funds for medication co-financing. Implementation is currently paused at Semuto Health Center IV amid larger funding disruptions and associated restructuring.

### Implementation Framework Development

**FIGURE 1:**
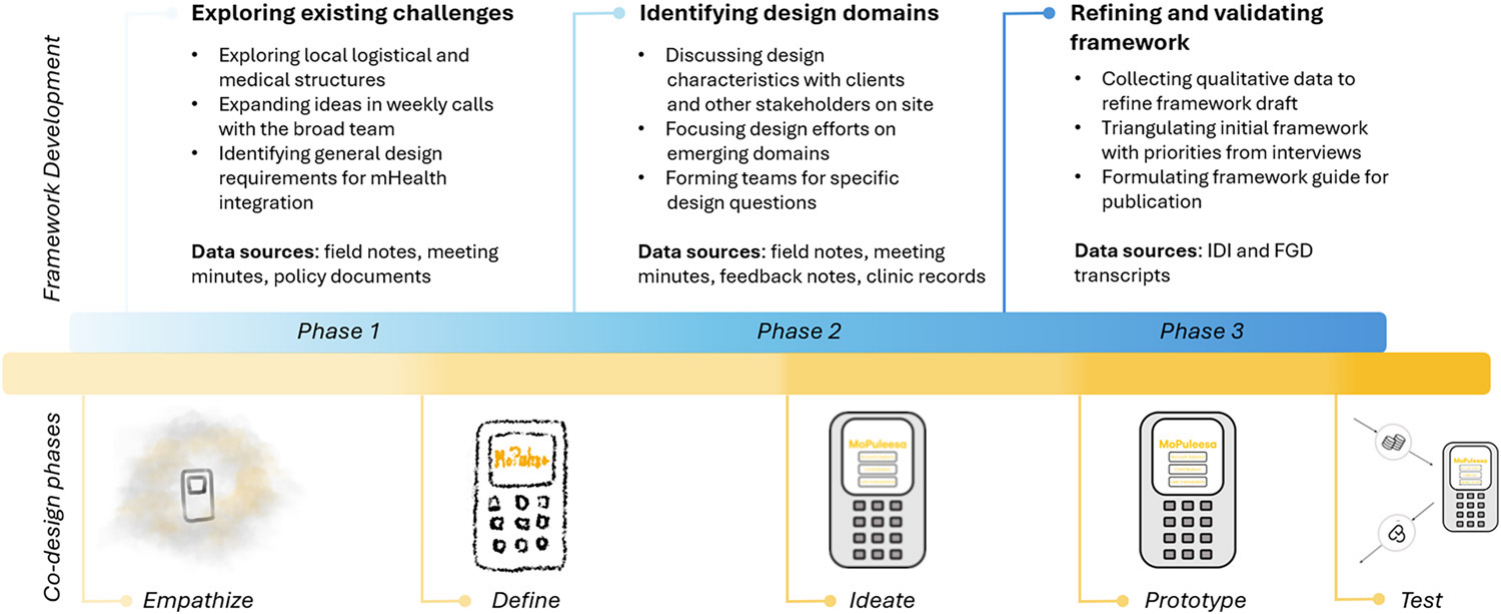
Framework Development Steps Abbreviations: FGD, focus group discussion; IDI, in-depth interview.

Over the course of intervention co-design and stakeholder involvement, design constraints repeatedly emerged, often associated with conflicting priorities and feasibility challenges (for example, feature phone compatibility or telecommunication provider and network requirements). We thus revisited the mHealth design literature but noticed a gap in resources acknowledging specific challenges, which sparked the idea to systematically synthesize our own design and implementation insights over the course of 3 phases between November 2021 and April 2023 (Figure 1). The first two phases consisted of formative research and iterative discussions, both among the research team and with key stakeholders, and aimed at ideating and designing a first framework concept. In the third phase, we refined and validated this framework, drawing on insights from in-depth interviews with key informants. Specific methods employed in each of these phases are outlined in detail below.

#### Phase 1: Formative Research

The first phase of framework development, overlapping with the ‘Empathize’ phase of the co-design process, took about 8 months and began with formative work to understand the context and to define priorities based on pre-existing parameters and emerging pain points. Project members across partner institutions (including several members with extensive experience working in the study setting) explored existing difficulties in NCD management in Nakaseke district. Additionally, we conducted observations in the study setting and unstructured interviews with key stakeholders (e.g., district policymakers, health care providers, community health workers [CHWs], and client representatives). Stakeholders were purposively sampled based on input from team members affiliated with ACCESS Uganda and familiar with the setting.

To identify initial design framework components, we revisited data including meeting minutes (of both weekly team meetings and conversations with key stakeholders), field notes (of observations in the study setting and discussions with clients), and clinic records. Identified design challenges were documented and discussed with a focus on the acceptability of intervention ideas on a medical, logistical, and technical level.

#### Phase 2: Refinement of Flows and Design Domains

In the second phase, which took about 5 months, research team members, including software developers, social scientists, and ACCESS Uganda representatives, formally involved prospective clients and other stakeholders in discussions on intervention and implementation design. Insights gathered from these discussions were documented in field notes and meeting minutes, helping us to clarify and refine intervention flows. We also analyzed existing infrastructure (e.g., electronic medical records) to identify potential synergies in the context of intervention implementation and testing.

Throughout this process, several specific challenges emerged within each of the general design dimensions observed in the first phase. Regular team discussions often led to the creation of specialized sub-groups for further iterations and decision-making (e.g., technical aspects such as functionality of intervention features were addressed by the software development team together with ACCESS Uganda; medical aspects such as treatment guidelines were discussed by medical professionals within the research team on-site). Over the course of this phase, we revisited and structured documented challenges and design decisions, resulting in the identification of 7 core domains that formed a first version of our framework.

#### Phase 3: Refinement and Validation of the Framework

With a first framework version at hand, we collected qualitative data to allow for further refinement. To collect detailed stakeholder insights, we conducted 37 in-depth interviews and 2 focus group discussions with clients visiting the health facility for hypertension treatment, 2 focus group discussions with local CHWs, 2 in-depth interviews with health care providers working at the health facility, and 16 in-depth interviews with local leaders and policymakers at district and national levels. Clients’ interviews were conducted in person on-site after research team members approached clients on clinic days asking for their participation; other key stakeholders were contacted via phone. Respondents were selected using a combination of criterion sampling (approaching clients visiting the clinic on designated hypertension treatment days); purposive sampling (reaching out to clients with hypertension, local leaders, and policymakers identified by the local partner institutions as being relevant for the study objectives); and snowball sampling (building on referrals from health workers and stakeholders to identify additional clients with hypertension and key community figures).^20^ To be eligible for participation, respondents across all groups had to be at least 18 years of age and provide written informed consent.

The qualitative data collection team included experienced interviewers who worked together with local CHWs to conduct the in-depth interviews and focus group discussions. Semi-structured interview guides focused on experiences with hypertension diagnosis and treatment, barriers to health care, and intervention prototype feedback. Systematic debriefings were conducted after each day of data collection to gather insights on emerging challenges.^21^ Data were analyzed using NVivo 13 following a 5-step framework approach.^22^ Results were triangulated with the initial framework version to further bolster design insights, specify domain characteristics, and finalize the framework. (For further information on data collection and analysis in the qualitative phase, see the COREQ guideline included as Supplement 2.) Concluding this final phase of framework development, which took about 3 months, we clarified framework domain definitions, identified domain-specific design decisions, and ideated potential broader operationalizations.

### Client and Public Involvement

Clients and public stakeholders were involved throughout the design and development of both the intervention prototype and the design framework, including via focus group discussions and in-depth interviews. Their insights shaped key aspects of framework domains, including emerging challenges and preferred solutions, ensuring that clients and public priorities were incorporated. Details on stakeholder involvement within each study phase are outlined above.

### Ethical Considerations

This study received ethical approval from the Ethical Review Board of the Medical Faculty, Heidelberg University, Germany (Registration Nr. S-299/2022) and the Makerere University School of Biomedical Sciences Research and Ethics Committee (SBS-REC, Registration Nr. SBS-2022-175), Kampala, Uganda. The study has been registered with both the German Clinical Trials Register (DRKS: HS2445ES) and the Ugandan National Council of Science and Technology (HS2445ES).

## RESULTS

**FIGURE 2:**
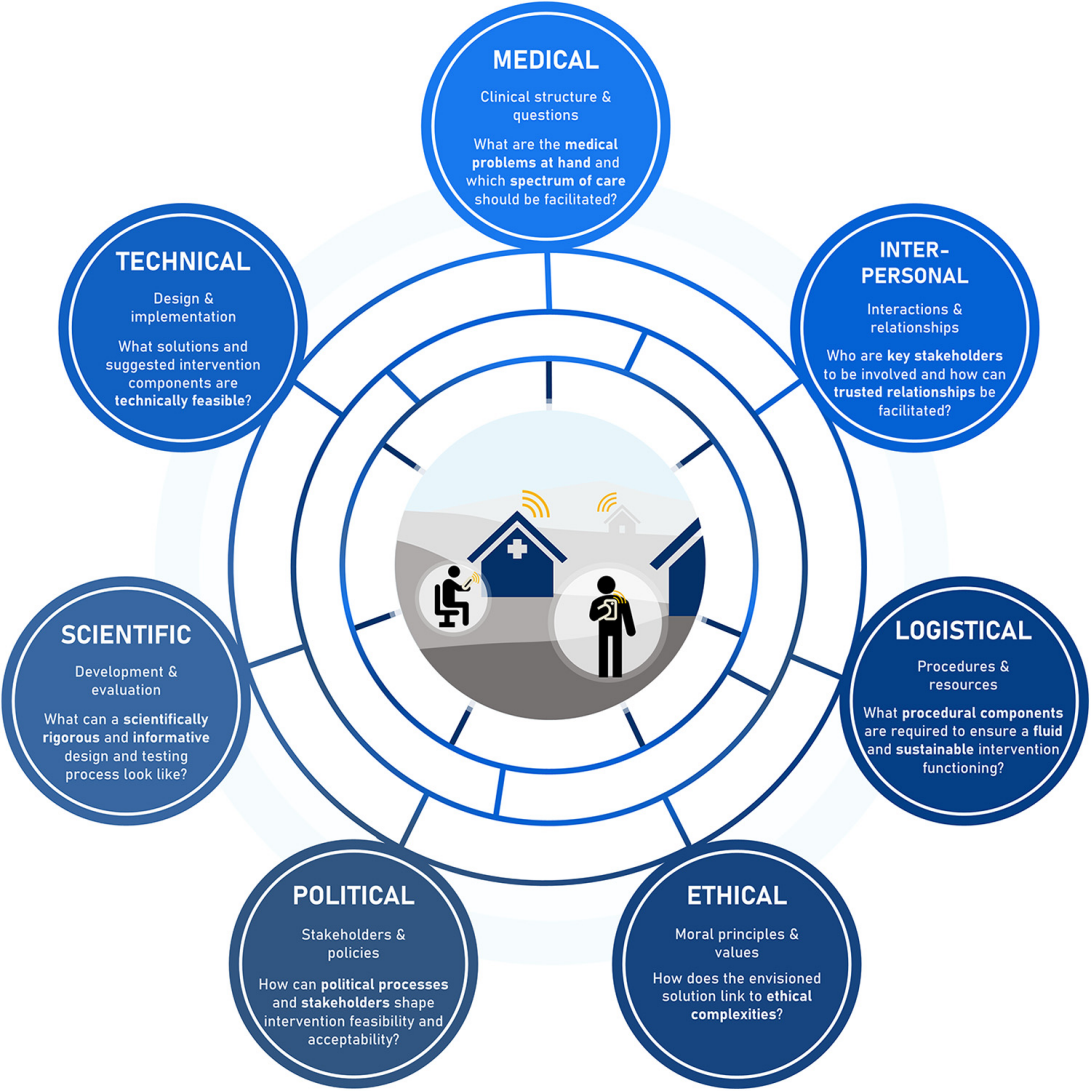
The MILEPOST Framework for Co-Designing mHealth Interventions

In this section, we outline the identified 7 design domains to consider when co-designing mHealth interventions, which together form the MILEPOST framework: medical, interpersonal, logistical, ethical, political, scientific, and technical (Figure 2). All domains, seemingly independent at the outset, are connected by an interlinked network of pathways. As design and implementation progress (moving toward the center of the structure), these connecting elements increase and merge insights across individual domains, reflecting the growing complexity of, and need for, cross-domain considerations. Nested at the center of the network is the community-based intervention prototype, signifying the overarching aim of an intervention that meets end-user expectations and priorities.

For each domain, we start by providing a definition of its scope. We then outline how this domain manifested in the form of specific challenges and associated design decisions in our own work. We conclude this section by summarizing the broader relevance of each domain and propose recommendations for operationalizing the MILEPOST framework by introducing a checklist that could guide future projects. For verbatim quotes, we use the respondent group (client with hypertension, CHW, health care provider or local leader/policymaker) as identifier. For clients, CHWs, and health care providers, we additionally include age and gender; for local leaders/policymakers, we provide more precise information on their role.

### Medical

The medical domain refers to considerations regarding medical practice in the specific local context (e.g., medical intervention end-goals, existing health system structures, or local clinic procedures and priorities).

Our early formative research highlighted the challenge of ensuring a sustainable and consistent supply of affordable hypertension medication. Although availability of all types of antihypertensive medication was repeatedly disrupted, stock-outs most often occurred for the two most prescribed medications: Bendroflumethiazide (a diuretic) and Nifedipine (a calcium channel blocker). Procuring these medications at private facilities costs clients 3,000 to 15,000 Ugandan Shilling (US$0.81 to $4.04) for 30 pills, which corresponds to 3% to 19% of the average monthly income in the study setting. This high cost forced many clients to stop “taking those meds, because they are so expensive” (Female CHW, age 50). One client with hypertension described how:

*We receive drugs, but most of the time, the drugs are not available. That even resulted in the death of my mother. We didn’t have money to buy drugs because they are so expensive. (Female client, age 62)*

Based on the identified medical challenge of frequent medication stock-outs and high out-of-pocket expenditures, we explored specific medical design decisions. These decisions included, for example, which types of medication to include in the intervention (based on setting-specific prescription frequency); whether/how to address potential comorbidities requiring comprehensive care (roughly 20% of the eligible clients had both diabetes and hypertension; however, to facilitate pilot implementation and testing, and based on cross-domain considerations, we only included antihypertensive medication for intervention piloting); and how to leverage and supplement existing clinical structures while minimizing intervention-associated disruption to routine care.

### Interpersonal

The interpersonal domain focuses on interactions and relationships between individuals, including preexisting interpersonal dynamics or social exchanges within and across team members, communities, and stakeholder groups.

In our work, we iteratively involved key stakeholders, including clients, health care providers, and policymakers. In these interactions, one of the most prominent pieces of design feedback was the importance of interpersonal trust and transparency. Concerns surfaced regarding integrating a complex mHealth component due to fears that this might result in a dependence on unknown or untrusted individuals, organizations, and technologies. Clients therefore frequently voiced a strong desire for committed and long-term intervention leadership:

*It needs to have a trusted person, so that the money is not collected and after two months they say that it got lost, the drugs were not bought. We need trust so that the drugs are bought and are there. (Male client, age 65)*

To acknowledge the importance of interpersonal relationships, we invested considerable effort in identifying trusted staff members and building rapport with end-users via repeated feedback rounds and transparent communication about intervention development processes and limitations. This, for example, involved the project coordinator or a CHW whom clients already knew introducing all data collection and study activities in person at the health facility.

Our results also highlighted how clients often relied on relatives to pool funds for their health care costs:

*I have a son who cares for me financially because I don’t have any other job. (Male client, age 62)*

Emphasizing the relevance of the interpersonal domain regarding functional aspects of the intervention itself, this observation informed our design decision to include features for next-of-kin to directly contribute funds on behalf of the client (see also technical considerations). At the same time, some clients also expressed the wish for an elected spokesperson to coordinate the saving scheme among participants and relay feedback to the project coordinator. In our pilot implementation phase, however, scheme and intervention features were not yet final, and rapid communication channels between implementation and development teams were crucial. Therefore, based on cross-domain considerations, this client priority was not realized in the first intervention iteration but is being considered for post-implementation adaptation.

The interpersonal domain also manifested within our multidisciplinary team comprising members involved in all stages of creating, using, and evaluating the mHealth intervention. Within the broader team calls, competing priorities emerged over the course of the project, for example, when balancing technical feasibility versus ideal medical properties, trial-based testing versus rapid and iterative post-implementation refinement, or decisions on funding allocation. We therefore aimed to facilitate transparent communication and established regular platforms for open discussions across disciplines and participating organizations. We also used openly editable documents (Miro Boards) to collect and discuss emerging questions.

### Logistical

The logistical domain entails processes relevant for intervention design and implementation, including available resources, key environmental and societal factors, and large-scale infrastructure.

With our work focusing on the integration of mHealth components into a preexisting paper-based system, logistical challenges during prototype development often linked to technical considerations. Such challenges, for example, included the need for shared client identifiers allowing linkages between platform contributions and clinical records; see also considerations in the technical domain. These difficulties required acknowledgment early in the design process to avoid technical revisions at later stages. To rapidly clarify logistical questions, we established direct communication channels linking local stakeholders with technical leads, and we developed and iteratively refined detailed logistical road maps that visualized all intervention processes.

However, when implementing the prototype, logistical challenges moved beyond the mHealth component itself. One such newly emerging obstacle, linking logistical and interpersonal domains, was the explicit desire for clear storage and accounting procedures, as well as for a strict separation of all intervention-related monetary transactions from regular clinic procedures to maintain trust and transparency:

*It’s all about that, proper requisition books, main store, a procured cupboard for storage, to have a focal person concerned about that. (Female nurse, age 36)*

Responding to these requirements, we aimed at ensuring separate drug distribution points and processes for government and intervention-based supplies. Another challenge was the need for an accessible but confidential two-step system for eligibility assessment and drug disbursement, which maintained clear separation between the government-funded and the intervention-based medication supply (see also ethical considerations below). The need for such clear separation to alleviate legal concerns was highlighted by a health care provider involved in the original community-initiated scheme who, in light of challenges to prove that she was not trying to charge money for government-funded drugs, “stopped [distributing the medication] before they imprison me” (female nurse, age 36).

Desire for a sustainable intervention, shared by both study team members and local key stakeholders, permeated logistical design considerations. Facilitating this sustainability required an in-depth understanding of the (logistical) situation pre-implementation and iterative adaptations across design phases and MILEPOST domains. Outlining the need for design decisions that would allow for timely and first-hand observation of contribution benefits, one client highlighted:

*If the platform is a success, people will greatly use it, but if they come when the medicine is not available and we are told to go and buy, yet we already saved, it will discourage people. (Female client, age 47)*

### Ethical

The ethical domain includes design decisions relating to ethical values and priorities underpinning both the intervention itself (e.g., equity, responsibility, and accountability) and all research and data collection activities (e.g., privacy, confidentiality, and data protection).

In our study, designing an intervention that links medication to individual-level financial contributions posed an ethical challenge, especially in the context of a health care system meant to be free of cost. One scenario repeatedly discussed within and beyond the research team was whether to deny medication available in the intervention pool to clients who did not contribute (or who were not able to meet the envisioned minimum contribution):

*The disadvantage is that there are those who can’t afford because there are some poor people here in the community who cannot even earn 10 dollars a year. (Male clinician, age 56)*

Linking to the interpersonal dimension, clients furthermore emphasized the importance of trust and reliability in the context of a research pilot project that fosters hope, reflecting on previous systemic disappointments:

*A problem will arise if the treatment is paid for and they tell us that the drugs are not there, just as the [government] system has been. (Female client, age 40)*

Across design decisions in our study, we acknowledged the sensitive nature of these ethical considerations by placing particular emphasis on ensuring and moving beyond the formal requirements set by institutional review boards and international agreements and declarations. Regarding concerns associated with individual-level payments for treatment access, we followed the flow and rationale of the preexisting community-initiated intervention. We also continued to rely on the strengths of participatory and qualitative approaches to iteratively evaluate our design decisions and to incorporate end-user feedback. This approach aimed to address stakeholders’ concerns that our work might inadvertently result in another well-intended but ultimately counterproductive intervention:

*We shall need proper management of funds because most times people come up with programs but the money ends up in the wrong hands. (Female CHW, age 38)*

However, this iterative process also included instances where feedback conflicted with other design priorities or was technically and logistically infeasible. This, for example, included the request for “quarterly reviews” meant to “help us to monitor the project and within the review, we’ll need the stakeholders to give feedback” (female nurse, age 36). While the research team and other stakeholders agreed that systematic review and feedback loops were essential, we ultimately opted for addressing emerging implementation issues on a rolling basis (to maintain flexibility) and conducting post-piloting feedback interviews (for systematic process and intervention evaluation); more formalized review intervals are being currently explored by project partners leading post-study implementation and maintenance.

### Political

The political domain focuses on intervention alignment with local policies and regulations, as well as the integration of policymakers’ insights into implementation processes.

ACCESS Uganda, over the years of its engagement in this setting, had established trusted partnerships with local governing bodies. Building on these experiences and insights, and to facilitate potential scale-up following proof-of-concept, we closely collaborated with policymakers from study outset. Over the course of this collaboration, the idea to introduce a financing scheme in a government health facility initially sparked concerns, questions, and recommendations on the political level:

*It shouldn’t be managed by the patients because when the health workers are involved then the politicians will come for them. It will be seen as an extortion because the services are free, and then you put their nurse to collect money from patients who come. That will be seen as extortion because you are charging for a service which is supposed to be free. (Male policymaker, national level)*

While echoing these challenges, local policymakers and health care providers also shared experiences and concerns regarding potential conflicts or hidden motives involving local and national political stakeholders:

*There is a group of politicians who have their own selfish agendas. They always come with different goals to climb in leadership. It’s not good to involve them. (Male policymaker, local level)*

To respond to political impediments, we iteratively collected and integrated feedback from policymakers at local and national levels within formative and qualitative research phases. To facilitate the inclusion of policymakers and local stakeholders beyond the original network, we used snowball sampling based on interviewee recommendations (linking to the scientific domain). Furthermore, recognizing the importance of fostering transparency and collaboration, we secured additional funding to engage individual policymakers before and during intervention introduction and to hold dissemination workshops to communicate experiences and challenges voiced by participants following prototype introduction. Beyond outlining the community-driven and collaborative nature of intervention development, these workshops aimed at co-developing actionable strategies for long-term implementation and sustainability. Through this stakeholder engagement, we aimed to create a shared vision for intervention management that emphasized collaboration, inclusivity, and alignment with diverse stakeholder perspectives.

### Scientific

The scientific domain encompasses all considerations regarding rigorous and comprehensive prototype design and testing, combining established best-practice approaches and novel methods.

In our project, scientific challenges emerged when working with inconsistent or hard-to-access data. Notable examples included variations in client numbers accessing the clinic (impeding reliable power estimates) and the need for one platform allowing to trace individual participants across different datasets (e.g., clinic utilization, platform usage, and medical records). Additionally, scientific-methodological challenges emerged during the quantitative pilot-testing of the intervention prototype. These challenges included the identification of meaningful but measurable outcomes, the user-specific longer-term tracking of platform engagement and medication pickup, and the development of comparators and randomization strategies that balanced logistical demands, ethical considerations, and scientific rigor.

We addressed emerging obstacles by gathering setting-specific information from various sources, bringing together trialists, technical experts, qualitative design researchers, and stakeholders with setting expertise. Additionally, we continuously refined key scientific markers such as respondent groups, sampling frames, and data collection tools. We engaged local CHWs as research assistants and recruitment coordinators, which facilitated trust and openness in research participants but also required additional training (to ensure data quality) and process refinement (to avoid clinic flow disruptions). We also obtained additional funding to expand the local team of quantitative and qualitative researchers, to extend piloting and refinement of data collection instruments, and to make existing resources accessible for design and evaluation activities (e.g., assessing available clinic records, both digital and analogue, with the support of additional staff). For the testing phase, we developed two intervention prototypes to allow for meaningful comparison without barring interested clients from continued contribution to the pooled financing scheme (balancing scientific, logistical, and ethical dimensions). We also adjusted quantitative data collection approaches based on end-user feedback to improve speed and minimize waiting times while maintaining data quality.

### Technical

The technical domain in the context of mHealth interventions includes hardware and software components, user-interface design, and data management.

In our study, the mHealth intervention built on a preexisting community-initiated system, which required fundamental revisions of available software infrastructure and financing flows. These revisions sparked technical challenges when aligning stakeholder feedback and scientific expectations with technical feasibility. One design demand was for our application to be compatible with standard feature phones rather than smartphones to ensure accessibility in light of the available network, hardware, and familiarity (especially of elderly clients). Furthermore, our HCD approach repeatedly sparked design ideas that, from a technical perspective, could not be rapidly iterated in a working prototype (including, for example, the possibility for next-of-kin contributions, reminders, or contributions across various phones). Additional technical challenges, in line with logistical and sustainability considerations, were the engagement of local third-party telecommunication service providers (led in tandem by local and technical teams), as well as the need for longer-term technical support, especially onboarding new clients to the platform beyond project funding. This was also emphasized by clients:

*If you start, you need to educate our people. Like me, you first took me through [the system]. If we get to be educated about that thing, everything will be okay. (Male client, age 60)*

Technical development of the prototype only commenced after the first phase of qualitative data collection and prototype ideation had concluded. Nevertheless, trade-offs were required between the flexible integration of end-user feedback and technical development cycles, which we tried to address by bundling insights within research phases to determine the broad technical structure and, where possible, limit design revisions prior to pilot-testing to a manageable level. Additionally, technical, scientific, and medical teams closely collaborated to identify actionable compromises such as, for example, adjusting intervention implementation when one telecom provider changed the prices for cash deposits (which disrupted contributions, drug purchasing, and clinic flows).

### Broader Relevance and Recommendations

While specific design experiences and decisions might vary across projects, we consider each MILEPOST domain to be relevant for a wide range of projects aiming to develop meaningful and useful interventions. The Table outlines how domains might manifest across different co-design efforts. At the same time, as highlighted in our experiences, many challenges might emerge at intersections between several MILEPOST domains. These tensions underscore a need for iterative, collaborative processes not only in intervention and implementation design but also in team communication, decision-making, and general study procedures.

**TABLE.** Broader Relevance and Potential Manifestation of MILEPOST Domains Across Different mHealth Interventions

Domain	Relevance
**Medical:** *Main focus, existing structures, and practice*	Likely a priority of most design projects given the field of global health’s inherent focus. Specific challenges might differ substantially across projects (e.g., while chronic disease management focuses on long-term sustainability and comorbidities, prevention efforts might prioritize client education and at-home usability).
**Interpersonal:** *Social interactions and relationships, within and across intended populations*	Crucial across settings and teams, especially regular and transparent communication. Variations in preexisting trust, timelines, or controversies across projects might require leveraging specific forms of expertise and refining communication channels.
**Logistical:** *Existing and required resources, infrastructure, and environment*	Requires careful assessment, particularly regarding (long-term) infrastructural needs to facilitate mHealth intervention sustainability. Projects might differ in terms of specific logistical demands and resource requirements, e.g. depending on end-users (provider vs. patient-facing) or delivery pathways (at-home, outpatient, or inpatient).
**Ethical:** *Equity, responsibility, and accountability*	Central but often complex, highly context-dependent, and hard to navigate amid medical urgency, donor demands, and requirements from ethical review boards. Careful consideration of ethical dimensions and adherence to existing best practices must guide all design decisions.
**Political:** *Local policies and dynamics, including barriers and facilitators for intervention rollout*	Involves acknowledging and navigating (often conflicting) interests of a broad range of stakeholders, also depending on medical focus (e.g., addressing mental or sexual health might be more controversial than a cardiovascular disease focus). Early involvement of political and community stakeholders and their priorities can facilitate intervention success and sustainability.
**Scientific:** *Rigor, relevance, reliability, and validity*	Associated challenges are shaped by research focus, employed approaches, or data sources (e.g., digital usability data contributes different information than qualitative end-user insights). Open-ended exploration of various approaches’ feasibility can facilitate scientifically rigorous assessments without compromising other priorities.
**Technical:** *Hardware and software, user-interface, and data management*	Requires early and iterative assessments of technical requirements and feasibility to guide design idea prioritization, to avoid end-user disappointments, and to safeguard technical resources. May overlap with logistical concerns (e.g., distribution systems or human resource needs).

Supplement 3 further expands on this expected broader relevance by providing specific starting points for operationalizing MILEPOST in future design work. We define each domain and outline key questions that may aid researchers and designers in developing an understanding of how each domain manifests in their specific context. Additionally, we outline potential stakeholders (within and beyond study teams and intended populations) who, based on our experience, could serve as first points of contact for exploring these questions. These guidelines are by no means intended to be exhaustive; each project and context will introduce unique guardrails, challenges, and key considerations. We advocate for flexible adaptation, expansion, refinement, or redaction of these guidelines to suit specific project needs.

## DISCUSSION

Based on challenges encountered when co-designing an mHealth intervention in rural Uganda, this article maps out 7 design domains to consider when co-designing mHealth interventions that together form the MILEPOST framework: medical, interpersonal, logistical, ethical, political, scientific, and technical. We outlined how these domains emerged in our work, how we addressed associated challenges in practical design decisions, and how domains could manifest and be operationalized in other studies or settings.

With the identification of these specific domains, MILEPOST links to an ongoing discourse on how to design appropriate interventions. We echo existing scholarship that highlights both the importance and the potential of participatory design using mixed-method approaches and that emphasizes the need to share and learn from experiences when designing and implementing complex interventions in specific settings.^23^^,^^24^ In its overarching aim, MILEPOST thereby aligns with existing design scholarship including step-by-step-approaches,^25^ questionnaires,^26^ and structured synopses^27^ covering use-cases from physical activity interventions^28^ to individual-level decision-making support.^29^ Many of these design guidelines and frameworks refer to complex and abstract constructs, such as social and behavior change theories^30^ (e.g., the Health Belief Model,^31^ the Theory of Planned Behavior^32^ or HCD^33^), which are particularly helpful when developing hypotheses but are often difficult to transfer into practical design decisions.^34^ While their focus on such general concepts makes many frameworks applicable to a broad array of intervention contexts, their direct practical application, in our experience, remains challenging due to limited operational suggestions and clear examples.^35^

At the same time, some frameworks aim to mediate between these abstract constructs and specific design guidance and experiences. One example is a framework following a “waterfall process”, which aligns with our aim to provide real-life examples for applied decision-making.^25^ However, this framework differs from MILEPOST by conceptualizing a sequential design approach, where each domain informs the next, and less focus on specific design challenges.^25^ In two more notable framework examples, both Mohr and colleagues^36^ (in their application of a Behavioral Intervention Technology Model) and Gurupur and Wan^37^ (in identifying 5 key challenges for mHealth interventions) outline practical examples for design guidance but mostly prioritize technical aspects.

In addition to these broader frameworks, individual facets of the MILEPOST domains are also mirrored in existing design literature: The scientific domain aligns with guidance in several more procedurally oriented design frameworks, such as recommendations to ensure carefully tailored outcomes,^25^^,^^30^ methodological considerations with a focus on mixed methods,^38^ or calls for flexibility when navigating conflicts between stakeholder ideas and scientific best practices.^39^ Our ethical domain links to considerations in Kass’s ethical framework for public health and its emphasis on potential intervention burdens^40^ (in our case, predominantly observed with regard to financial concerns). Trust and ownership, incorporated particularly in our logistical and interpersonal domains, also emerged in design work emphasizing the need for privacy and anonymized participation^41^ and in a systematic review of mHealth intervention features with a focus on credibility.^42^

In terms of management and uptake, Poland’s approach to understanding settings^26^ describes the importance of power relations and stakeholder interests as essential for comprehending the preconditions for an intervention, thereby linking to MILEPOST’s political domain. Additionally, key aspects emerging in our technical domain resonate with observations regarding the benefits and barriers of digital technologies.^27^ Finally, a 2022 systematic review on behavioral science and design thinking also identified challenges aligning with our medical (e.g., when introducing mHealth in a clinical setting), interpersonal (e.g., when involving key stakeholders), logistical (e.g., when adhering to specific time frames), and technical (e.g., when translating ideas into intervention features) domains.^43^ MILEPOST further builds on these existing insights by outlining how domains coexist (and often coincide) in design efforts, creating unique challenges at domain intersections that require setting-specific solutions.

Creating a framework such as MILEPOST cannot bypass the complex question of meaningful community engagement and sustainability in global health design efforts. Engagement and sustainability have been repeatedly argued to represent core components in transcending initial implementation and ensuring long-term intervention relevance.^44^^–^^47^ These core components are also reflected in arguments for resource and financing considerations^48^ and the need for procedural adaptability to changing circumstances and contexts.^49^ These arguments are echoed in our work, where the importance of community engagement and sustainability emerged repeatedly across domains.

Summarizing and categorizing recurring design challenges in the form of a framework provided us with a structured approach for understanding and addressing obstacles encountered throughout our participatory design work and allowed us to streamline intervention sustainability efforts. The ongoing implementation of the intervention prototype in the setting, more than one year after external funding has ceased, suggests at least an initial success. We hope that our described approaches can contribute to the literature on how to build interventions in a deliberate and collaborative fashion aimed at longer-term intervention utility.

### Limitations

While we aimed to outline a framework that can guide mHealth intervention designers and implementors more broadly, the MILEPOST framework was developed based on a specific study in rural Uganda. We see a particular strength in providing concrete examples for challenges and decision-making processes; nevertheless, this focus might also limit generalizability to other settings, health issues, intended audiences, or types of mHealth interventions. Additionally, as of this writing, the MILEPOST framework has not been systematically tested, and while the resulting intervention has shown promise in the context of initial implementation, longer-term and larger-scale insights into the framework’s significance in facilitating such successes remain limited. We therefore encourage future studies that evaluate and compare the acceptability, effectiveness, and sustainability of MILEPOST-informed interventions to provide additional insights into the applicability of the framework across settings. We also call for researchers and practitioners drawing on the MILEPOST framework to share their experiences and observations to gain a shared understanding of the framework’s potentials, shortcomings, and required refinements.

## CONCLUSION

Designing and implementing mHealth interventions requires a comprehensive and structured approach to ensure effectiveness, feasibility, and uptake. Based on our experiences when co-developing an mHealth intervention in rural Uganda, we call on designers and implementors of such interventions to consider medical, interpersonal, logistical, ethical, political, scientific, and technical dimensions of intervention development. We emphasize the importance of recognizing and addressing interactions and potential tensions between these domains to foster effective communication and collaboration within multidisciplinary teams. By providing practical insights across a range of design domains, we hope to support stakeholders, including researchers, developers, and policymakers, in their efforts to design successful and contextually aware mHealth interventions. We encourage future research on the potential of this framework and its applicability in other settings.

## Notes

**Peer Reviewed**
